# Dual-Mode Flexible Pressure Sensor Based on Ionic Electronic and Piezoelectric Coupling Mechanism Enables Dynamic and Static Full-Domain Stress Response

**DOI:** 10.3390/mi16091018

**Published:** 2025-09-03

**Authors:** Yue Ouyang, Shunqiang Huang, Zekai Huang, Shengyu Wu, Xin Wang, Sheng Chen, Haiyan Zhang, Zhuoqing Yang, Mengran Liu, Libo Gao

**Affiliations:** 1 School of Electronic Science and Engineering (National Model Microelectronics College), Xiamen University, Xiamen 361102, China37120222203456@stu.xmu.edu.cn (S.W.);; 2 School of Mechanical Engineering, Hubei University of Technology, Wuhan 430068, China; 3 Pen-Tung Sah Institute of Micro-Nano Science and Technology, Xiamen University, Xiamen 361102, Chinawangxin1@stu.xmu.edu.cn (X.W.); 4 Science and Technology on Vacuum Technology and Physics Laboratory, Lanzhou Institute of Physics, Chinese Academy of Space Technology, Lanzhou 730000, China; 5 National Key Laboratory of Advanced Micro and Nano Manufacture Technology, Shanghai Jiao Tong University (SJTU), Shanghai 200240, China; yzhuoqing@sjtu.edu.cn

**Keywords:** flexible pressure sensors, dynamic and static forces, MWCNT composites

## Abstract

Flexible pressure sensors have shown promise applications in scenarios such as robotic tactile sensing due to their excellent sensitivity and linearity. However, the realization of flexible pressure sensors with both static and dynamic response capabilities still face significant challenges due to the properties of the sensing materials themselves. In this study, we propose a flexible pressure sensor that integrates piezoelectric and ionic capacitance mechanisms for full-domain response detection of dynamic and static forces: a “sandwich” sensing structure is constructed by printing a mixture of multi-walled carbon nanotubes (MWCNTs) onto the surface of the upper and lower electrodes, and sandwiching a polyvinylidene fluoride (PVDF) thin film between the electrodes. The device exhibits a sensitivity of 0.13 kPa^−1^ in the pressure range of 0–150 kPa. The sensor has a rapid dynamic response (response time 19 ms/12 ms) with a sensitivity of 0.49 mV kPa^−1^ based on the piezoelectric mechanism and a linearity of 0.9981 based on the ionic capacitance mechanism. The device maintains good response stability under the ball impact test, further validating its potential application in static/dynamic composite force monitoring scenarios.

## 1. Introduction

In recent years, flexible pressure sensors have developed and have been widely used in cutting-edge fields such as electronic skin [1,2,3], health monitoring [4], human computer interaction [5], and soft robotics [6]. Due to their ultra-low modulus, high mechanical ductility, and excellent interface compliance, flexible pressure sensors are able to achieve stable signal outputs under complex deformation conditions, and they have gradually become one of the core functional modules in wearable smart devices [7,8]. However, despite the important progress made in regard to static force [9] and dynamic force [10], the realization of flexible sensors with both static and dynamic pressure responses still faces many challenges. Therefore, the development of flexible pressure sensors with a high response speed, wide bandwidth operation capability and environmental adaptability is of great significance in order to improve the accuracy and practicality of force sensing.

Currently, most of the research in this field has focused on the development of devices based on a single sensing mechanism, resulting in significant functional limitations. Piezoelectric sensors [11,12,13,14] struggle to detect static signals. For example, the sensitivity of piezoelectric devices based on CNT/PVA/ZnO composite materials [15] is only 0.259 mV·kPa^−1^, while sensors [16] made using glycine chitosan films have a sensitivity as low as 0.13 mV·kPa^−1^, neither of which can respond to static pressure. Although some studies have achieved stable piezoelectric responses at high temperatures using single-crystal AlN materials [17], they still cannot detect static forces, due to the lack of an integrated capacitive mechanism. Capacitive sensors [18,19,20,21] can detect both dynamic and static pressures simultaneously, but their sensitivity is generally low, typically ranging from 0.01 to 0.11 kPa^−1^, and cannot meet the requirements for high-precision measurements. For example, the sensitivity of P-PTC sensors, prepared via chemical vapor deposition (CVD) [22], is 0.080 kPa^−1^, with relatively long response times (0.48 s/0.62 s) and low linearity (0.54); a PDMS dielectric layer device, optimized with biomimetic microstructures [23], achieves a sensitivity of only 0.055 kPa^−1^ sensitivity; while a wireless capacitive sensor based on porous PDMS [24], has an even lower sensitivity, of 0.046 kPa^−1^. These devices, which do not use piezoelectric materials, have limitations in regard to their dynamic response performance. Overall, existing single-mode sensors typically face challenges in regard to achieving the synergistic optimization of multiple performance metrics in practical applications, such as simultaneously achieving high sensitivity, low signal delay, and good stability across a wide response range [25,26]. Especially in complex scenarios that require the simultaneous capturing of weak static contact and sudden dynamic impacts, such as robot perception [27,28], health monitoring, and wearable devices [29,30,31,32,33], the comprehensive sensing capabilities of such sensors are significantly inadequate. Therefore, developing a novel flexible sensor system capable of simultaneously responding to static and dynamic pressures, while also features a relatively wide response range, stable sensitivity, and fast response characteristics, has become an urgent need in order to drive the development of multi-modal intelligent sensing technology.

Herein, a flexible pressure sensor incorporating dual sensing mechanisms ionic capacitance and piezoelectricity is proposed. The device involves a composite sensing structure, which is created by screen printing an ionic solution containing multi-walled carbon nanotubes (MWCNTs) onto the surface of the upper and lower flexible electrodes, and that are sandwiched between the electrodes by a polarization-treated polyvinylidene fluoride (PVDF) film. Among them, the introduction of MWCNTs significantly improves the efficiency of the ion conduction path, thus accelerating the response of the sensor. The sensor shows a sensitivity of 0.13 kPa^−1^ and a linearity of R^2^ = 0.9981 over 0–150 kPa. The response and recovery times reached 19 ms and 12 ms, a sensitivity of 0.49 mV kPa^−1^ and the detection limit is 0.001 N in the piezoelectric mode of operation, and 33.4 ms and 58.7 ms in the ionic capacitance mode. The sensors exhibit good response performance in the face of both sudden shocks and steady-state pressure inputs, demonstrating the design’s broad applicability in regard to dynamic and static pressure monitoring. This work validates the feasibility and effectiveness of applying coupled ionic and piezoelectric mechanisms to flexible sensors, showing great potential for full-domain pressure response detection applications.

## 2. Materials and Methods

### 2.1. Materials

TPU particles were purchased from Crestron Polymers (Shanghai) Co., Ltd. (Shanghai, China) DMF and ionic liquids were purchased from Aladdin Reagent (Shanghai) Co., Ltd. (Shanghai, China) 95% MWCNTs were purchased from Suzhou Carbon Rich Graphene Technology Co., Ltd. (Suzhou, China) PVDF film was purchased from Polyk (Philipsburg, PA, USA).

### 2.2. Sensitive Layer Preparation Process

Dissolve 2.5 g of thermoplastic polyurethane (TPU) in 8 mL of N,N-dimethylformamide (DMF). Heat and stir under magnetic stirring at 100 °C until the TPU is completely dissolved to form a uniform transparent solution. After the solution cools naturally to room temperature, add 0.5 g of multi-walled carbon nanotubes (MWCNTs) and continue stirring at room temperature for 1 h to ensure thorough dispersion. Then, 3 mL of 1-ethyl-3-methylimidazolium bis(trifluoromethanesulfonyl)imide salt ([EMIM] [TFSI]) is slowly added dropwise during stirring, and stirring is continued for 30 min to obtain a uniform ionic solution.

### 2.3. Assembly of the Sensor Device

Using screen printing, the above ionic solution was uniformly printed onto the circular electrode in the center of the FPC and heated on a 60 °C heating plate until the solvent was removed, forming an ionic film. Subsequently, two FPC were bonded face-to-face along the edges of the FPC, and a PVDF film was placed between the upper and lower ionic films, forming a composite dielectric layer structure with the PVDF film and the ionic film on the electrode surface, resulting in the creation of the sensor.

### 2.4. Characterization and Measurement

The surface and cross-sectional structure of the sensor’s sensitive layer are characterized using a scanning electron microscope (SEM, Apreo 2S, Thermo Fisher Scientific, Waltham, MA, USA). The most critical part of sensor performance testing is signal collection under pressure. Sensor performance testing primarily consists of two parts: pressure loading and signal acquisition. Static pressure is applied by a pressure machine, and capacitance changes are measured by an LCR meter (TH2828A, Shanghai Tonghui Electronics Co., Ltd., Shanghai, China). During testing, the sensor sample is fixed to the pressure machine platform and connected to the LCR meter via wires. Pressure loading and data acquisition are controlled and recorded by a computer.

## 3. Results

### 3.1. Design and Application

**Figure 1:**
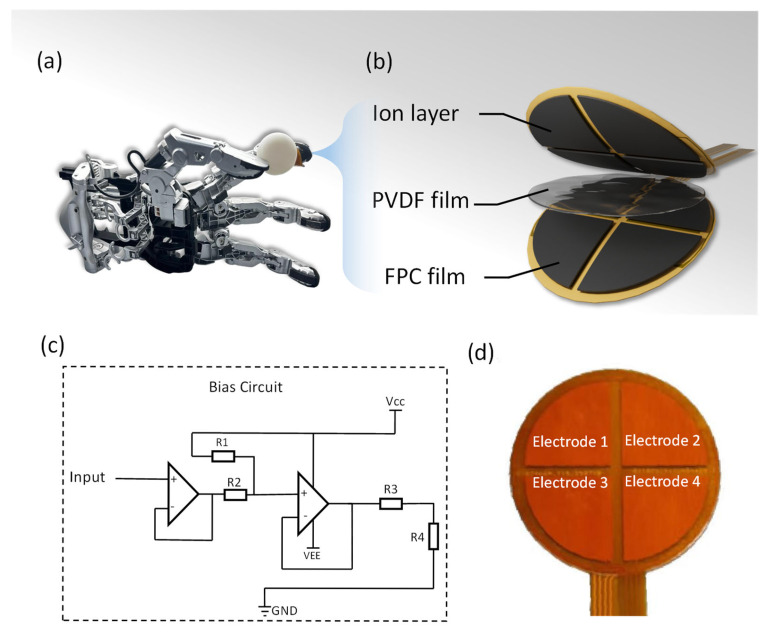
Schematic diagram and physical demonstration of flexible pressure sensor; (**a**) application of the sensor on a robotic arm; (**b**) schematic diagram of the sensor structure; (**c**) circuit framework diagram of the sensor; and (**d**) actual image of the sensor.

We experimentally fabricated a sensor combining ionic electronic and piezoelectric properties to detect both dynamic and static forces. This sensor can be attached to the tip of a robotic finger (Figure 1a) to monitor changes in both dynamic and static forces when the robotic hand comes into contact with an object. Figure 1b shows the structural diagram of the sensor, which adopts a “sandwich” layered design, from top to bottom: ion layer, PVDF film, and flexible printed circuit substrate (FPC film). The ion layer enhances the interfacial charge effect to improve signal output sensitivity; the PVDF film serves as the piezoelectric sensitive layer, responding to externally applied pressure signals; while the FPC layer serves both as an electrode and a support substrate, enabling signal extraction and flexible adhesion of the device.

Figure 1c shows the bias circuit for the sensor signal, consisting of two operational amplifiers and several resistors. The sensor output signal (input) is first input into the first operational amplifier, where R1 and R2 are used to set the gain, thereby performing preliminary amplification of the input signal; then, it passes through the second operational amplifier for further adjustment of the signal amplitude and bias, with R3 and R4 used to set the operating point and gain of the second amplifier. The circuit is connected to positive and negative power supplies (V_CC_ and V_EE_) at both ends to ensure the normal operation of the operational amplifiers. This circuit amplifies and adjusts the bias of the weak sensor output signal, facilitating subsequent data acquisition and processing. Figure 1c shows a physical diagram of a four-channel sensor. This sensor adopts a four-sector electrode configuration. This spatially discrete arrangement allows the sensor to independently capture local deformation information along different radial axes within the sensing plane. When an external stress field is applied to the sensitive material covering the electrodes, it induces differentiated responses in the regions beneath each sector electrode. The key point is that the angular displacement between the sector electrodes provides the sensor with inherent spatial resolution and stress direction sensing capabilities.

### 3.2. Sensing Mechanism

**Figure 2:**
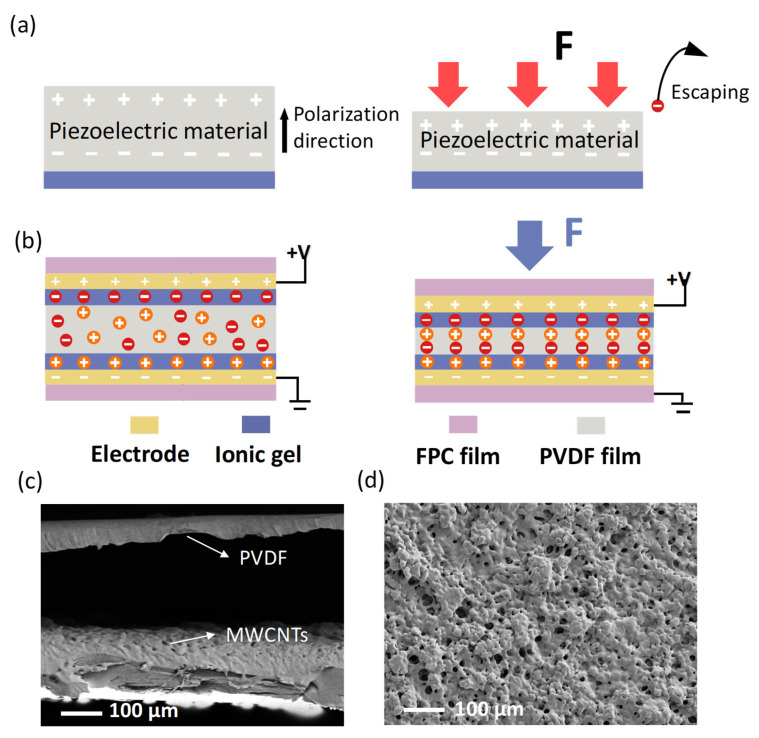
Mechanical schematic diagram and microscopic structure characterization of the sensor (**a**) schematic diagram of the piezoelectric effect; (**b**) sensing mechanism of the ionic double layer; (**c**) SEM screenshot of the sensor; and (**d**) SEM image of the surface resistance of the sensor.

The sensing mechanism of the sensor described in this paper is based on a combination of piezoelectric and iontronic effects. Figure 2a shows a schematic diagram of the piezoelectric effect. Under sustained pressure, ions in the piezoelectric material lose their double layer effect after polarization. When the sensor is subjected to a sustained, stable pressure, the ionic electronic effect plays a dominant role. After the pressure stabilizes, the deformation of the PVDF film no longer changes, and the instantaneous charge generated dissipates, as it is unable to sustain the output. Figure 2b illustrates the ionic effect. When the sensor is subjected to pressure, the ions in the sensitive layer rapidly adsorb onto the electrodes, forming a double layer to enhance capacitance.

The ionic double layer mechanism achieves pressure resolution at the sub-Pascal level, significantly expanding the sensor’s application range. The SEM cross-sectional view (Figure 2c) shows that there is a gap between the MWCNT layer and the PVDF film interface. This gap ensures the free deformation of the PVDF film under pressure, avoiding mechanical constraints on the piezoelectric effect caused by the rigid MWCNT network, thereby enhancing the sensor’s sensitivity. Additionally, the irregular distribution of the MWCNT network at the interface and within the material maximizes its exposed area, facilitating a stable signal output from the sensor. The SEM images of the printed conductive ink (Figure 2d) further confirm that the MWCNT are spread throughout the material, forming a porous MWCNT network. The introduction of MWCNT significantly improves the efficiency of ion conduction pathways, thereby enhancing the sensor’s sensitivity. This structural design provides the foundation for the sensor’s high sensitivity and low-noise output, mitigating noise interference caused by uneven conductive pathways. Through the synergistic design of the structure and material, the sensor can fully utilize the complementary advantages of piezoelectricity and capacitance to effectively detect dynamic and static stress fields.

### 3.3. Sensor Performance Characterization

Based on the performance provided by the above sensing mechanism, we conducted a detailed characterization of the sensor performance. The sensitivity (*S*) of a pressure sensor is an important indicator that measures the rate of change in the output signal when a unit of pressure is applied, directly determining the system’s ability to resolve micro-stresses.

(1) $$S=\frac{\delta \frac{\Delta C}{C_{0}}}{\delta P}$$

where *S* denotes sensitivity, Δ*C* denotes the change in capacitance under pressure, *C*_0_ denotes the initial capacitance before pressure application, and *P* denotes the applied pressure. Table 1 is a comparison table on the sensitivity of piezoelectric sensors.

**Figure 3:**
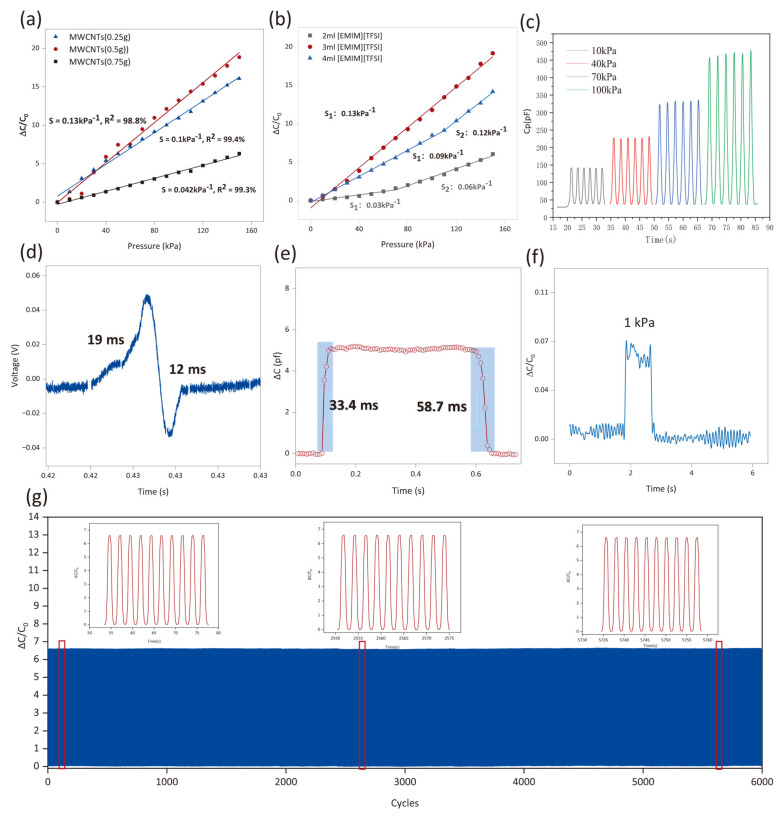
Performance characteristics of the sensor under different conditions: (**a**) exploring the optimal concentration of MWCNTs; (**b**) exploring the optimal concentration of ionic liquids; (**c**) stability testing of the sensor at 10 kPa, 40 kPa, 70 kPa, and 100 kPa; (**d**) response time and recovery time of the piezoelectric sensor; (**e**) response time and recovery time of the capacitive sensor; (**f**) the detection limit of the sensor; and (**g**) the stability of the sensor when it undergoes 2000 cycles at 50 kPa.

Figure 3 summarizes the characterization results under various experimental conditions. We investigated the effects of different concentrations of MWCNTs and ionic liquids on sensor performance and found that when the MWCNT mass was 0.5 g, and the [EMIM] [TFSI] is 3 mL, the sensor exhibits better sensitivity and linearity. The results show that under pressures ranging from 0 to 150 kPa, the sensor’s sensitivity reaches 0.13 kPa^−1^ and its linearity reaches 98.8%. Furthermore, we verified that the sensor exhibits a good response and low hysteresis under different pressures (Figure 3c). Based on the high response characteristics of the piezoelectric effect (Figure 3d), the sensor has an extremely fast response time (19 ms/12 ms), and in terms of electrostatic response, it also has a good response time (Figure 3e). Regarding long-term stability, the sensor exhibits a hysteresis error of only 1.2% after over 2000 cycles of 50 kPa pressure loading, demonstrating its exceptional mechanical durability. This stable capacitance change is primarily attributed to the reversible regulation capability of the ionic liquid interface double-layer structure under pressure.

The results indicate that at 0–150 kPa, the sensitivity of the MWCNT sensor at 0.5 g reaches 0.13 kPa^−1^, which is higher than the other two, and its linearity reaches 98.8% (Figure 3a). When 0.75 g of MWCNTs were added, the sensor’s sensitivity decreased because excessive MWCNTs were aggregated into micron-sized clusters due to van der Waals forces, significantly reducing the effective specific surface area and interfacial polarization density, leading to a significant attenuation in the relative change in the capacitance response (Δ*C*/*C*_0_) per unit of pressure change, Secondly, MWCNTs clusters filling the polymer matrix pores significantly increase the dielectric layer’s rigidity, weakening its deformation capability under pressure, resulting in a reduced response amplitude of the capacitance change (Δ*C*). Figure 3b shows a sensitivity comparison of the three different amounts of ionic liquids. The results indicate that as the pressure increases, the 3 mL ionic liquid exhibits a linear change pattern, while the 2 mL and 4 mL ionic liquids show a piecewise linear change. Excessive ionic liquids envelop MWCNTs, increasing the spacing and preventing direct contact between the MWCNTs, thereby inhibiting the expansion of the conductive grid [40].

We then tested the sensor’s stability at 10 kPa, 40 kPa, 70 kPa, and 100 kPa (Figure 3c). As the applied pressure increases, the amplitude of the capacitance response gradually increases. Within each pressure range, the capacitance signal clearly exhibited an increase and decrease during the pressure loading and unloading processes, and the response amplitudes and baselines across different pressure ranges were distinctly distinguishable, indicating that the sensor can accurately distinguish mechanical signals at different pressure levels. As shown in Figure 3d, the response time of the sensor based on the piezoelectric effect is 19 ms, and the recovery time is 12 ms. When based on the electrostatic effect, the response time of the sensor is 33.4 ms, and the recovery time is 58.7 ms (Figure 3e). When 4 mL of ionic liquid is added, the excess ionic liquid forms a double layer with reverse polarization charges under an electric field, counteracting the directed aggregation of positive and negative charges on the electrode surface, resulting in a decay in the increase in the effective capacitance change ΔC. Additionally, after the ionic mobility saturates, the conductivity increases, causing charge leakage, which further degrades the capacitance response characteristics. Notably, the detection limit of this sensor is “1 kPa” (Figure 3f). To further test the sensor’s stability, we conducted 2000 cycle tests at 50 kPa, as shown in Figure 3g, demonstrating stable recovery performance during loading and unloading processes.

### 3.4. Impact Detection

**Figure 4:**
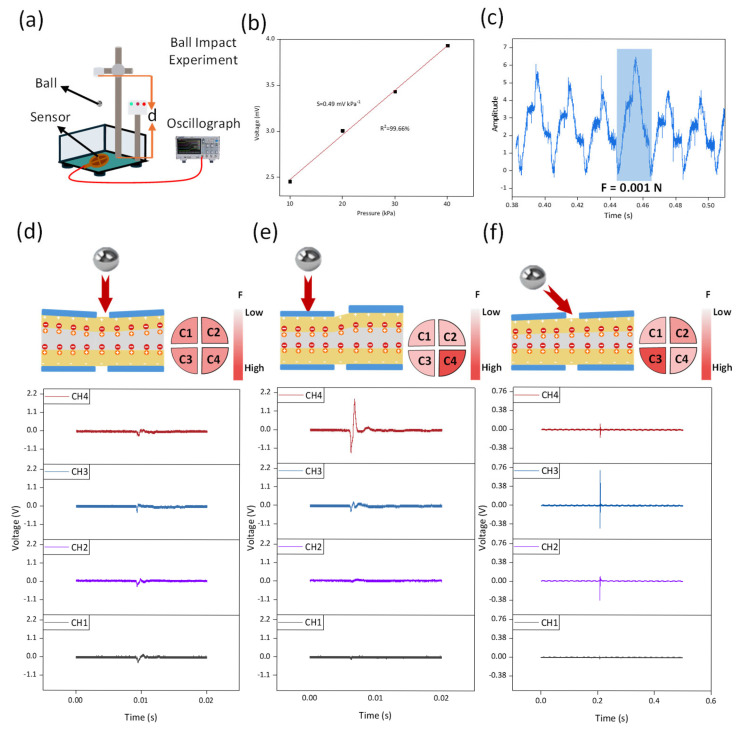
Sphere impact experiments and sensor responses at different impact angles (**a**) sphere impact experiment platform; (**b**) sensitivity of piezoelectric effect sensors; (**c**) detection limit based on piezoelectric effect sensors; (**d**) sensor response under a 90° sphere impact; (**e**) the response of the sensor’s single channel under a 90° spherical impact; and (**f**) sensor response under a 45° sphere impact.

The dynamic response performance of the sensor is characterized by impact detection. For this purpose, we constructed a ball impact test device (Figure 4a). This device uses DC electromagnetic control to allow the ball to fall freely. We tested the piezoelectric sensitivity of the sensor based on the piezoelectric effect, which was 0.49 mV kPa^−1^ (Figure 4b), and it was able to detect a minimum force of 0.001 N (Figure 4c).

Table 2 is a comparison table on the sensitivity of piezoelectric sensors. The ball used in this experiment weighs 3 g. We tested the ball impact response at 90° and 45°, as well as the single-channel impact response of the sensor at 90°. When the ball impacts the sensor surface, the impact force is converted into a three-dimensional force, detectable by the surface. The four sensor units distributed across the sensor array surface collectively invert this process, enabling the dynamic perception of the three-dimensional force. In the 90° and 45° ball impact experiments, the impact force was detected by all four sensor units. At 90°, the impact occurred at the sensor center (Figure 4d), and the responses of the four sensor units were found to be largely consistent. In regard to the 90° impact, one sensor unit (CH4) showed the smallest response toward channel 1 (CH1), while the adjacent CH2 and CH3 units exhibited lower responses than CH4 (Figure 4e). In regard to the 45° impact at the sensor center (Figure 4f), CH3 showed the largest response, CH2 and CH4 had similar responses, and CH1 had the smallest response, further validating the sensor array’s detection and sensing capabilities for three-dimensional forces.

## 4. Conclusions

In this study, we report on a flexible pressure sensor that integrates iontronic capacitive and piezoelectric effects within a single device structure. By optimizing the composition of MWCNTs (0.25 g, 0.5 g, 0.75 g) and ionic liquid (2 mL, 3 mL, 4 mL), the sensor achieved optimal performance with 0.5 g of MWCNTs and 3 mL of ionic liquid, yielding a maximum sensitivity of 0.13 kPa^−1^. This level of sensitivity is comparable to or higher than many reported flexible pressure sensors, which typically exhibit sensitivities in the range of 0.01–0.11 kPa^−1^, thereby highlighting the performance advantage of our design. The sensor demonstrated excellent linearity (R^2^ = 0.9981) over 0–150 kPa and maintained reliable performance after 2000 loading unloading cycles. Furthermore, the synergistic coupling of iontronic capacitive and piezoelectric mechanisms enables both a rapid response and high stability under static and dynamic pressure conditions. The tests under varying ball impact angles and constant pressure confirmed the sensor’s ability to maintain fast and stable responses. These results demonstrate the novelty of integrating iontronic capacitive and piezoelectric effects in a single flexible sensor and underline its potential for reliable detection of both dynamic and static forces, which is crucial for its structural optimization and practical applications.

## Figures and Tables

**Table 1:** Comparison of the sensitivity of related capacitive sensors.

No.	Dynamic Force	Static Force	Sensitivity	Refs
1	√	√	0.1 kPa^−1^	[17]
2	√	√	0.1034 kPa^−1^	[27]
3	√	√	0.0186 kPa^−1^	[33]
5	√	√	0.08 kPa^−1^	[34]
4	√	√	0.055 kPa^−1^	[35]
6	√	√	0.0125 kPa^−1^	[36]
7	√	√	0.03 kPa^−1^	[37]
8	√	√	0.046 kPa^−1^	[38]
9	√	√	0.1 kPa^−1^	[39]
10	√	√	0.13 kPa^−1^	This work

**Table 2:** Comparison of the sensitivity of related piezoelectric sensors.

No.	Dynamic Force	Static Force	Sensitivity	Refs
1	√	×	0.259 mV kPa^−1^	[15]
2	√	×	0.13 mV kPa^−1^	[16]
3	√	×	0.35 mV kPa^−1^	[22]
4	√	×	0.072 mV kPa^−1^	[23]
5	√	√	0.49 mV kPa^−1^	This work

## Data Availability

Data are contained within this article.
